# Prospective Observational Study of Incidence and Preventable Burden of Childhood Tuberculosis, Kenya

**DOI:** 10.3201/eid2403.170785

**Published:** 2018-03

**Authors:** Andrew J. Brent, Christopher Nyundo, Joyce Langat, Caroline Mulunda, Joshua Wambua, Evasius Bauni, Joyce Sande, Kate Park, Thomas N. Williams, Charles R.J. Newton, Michael Levin, J. Anthony G. Scott, on behalf of the KIDS TB Study Group

**Affiliations:** KEMRI–Wellcome Trust Research Programme, Kilifi, Kenya (A.J. Brent, C. Nyundo, J. Langat, C. Mulunda, J. Wambua, E. Bauni, T.N. Williams, C.R.J. Newton, J.A.G. Scott);; Oxford University Hospitals NHS Foundation Trust, Oxford, UK (A.J. Brent, K. Park);; University of Oxford, Oxford (A.J. Brent, C.R.J. Newton, J.A.G. Scott);; Imperial College London, London, UK (A.J. Brent, M. Levin);; Aga Khan University, Nairobi, Kenya (J. Sande);; London School of Hygiene and Tropical Medicine, London (J.A.G. Scott)

**Keywords:** tuberculosis and other mycobacteria, children, epidemiology, preventable burden, incidence, case detection rate, prevention, contact tracing, isoniazid, chemoprophylaxis, TB, Kenya, bacteria

Substantial progress has been made in the fight against tuberculosis (TB); however, new approaches are needed to achieve the current target set by the World Health Organization (WHO) to reduce TB incidence to 90% of 2016 levels by 2035 (*1*). A key element of WHO’s End TB Strategy is the prioritization of preventive treatment (*2*). However, the preventable burden of childhood TB has not been quantified in prospective epidemiologic studies, and globally, only an estimated 7% of eligible children received isoniazid chemoprophylaxis in 2015 (*1*).

Diagnosis of TB is more challenging in children than in adults (*3*). In low-resource settings, where TB burden is highest, diagnosis often relies on poorly validated clinical algorithms (*4*). As a result, adequate surveillance data are lacking, and published estimates of the global childhood TB burden vary widely (*1*,*5*–*11*). High-quality prospective data on the TB burden and case detection rate (CDR) in children are recognized priorities (*8*,*11*,*12*), and population-level data showing the preventable burden of childhood TB might reinforce the public health case for chemoprophylaxis in children. We designed the Kilifi Improving Diagnosis and Surveillance of Childhood TB (KIDS TB) Study to estimate the incidence, CDR, risk factors, and preventable burden of childhood TB in Kenya.

## Methods

### Study Sites

**Figure 1:**
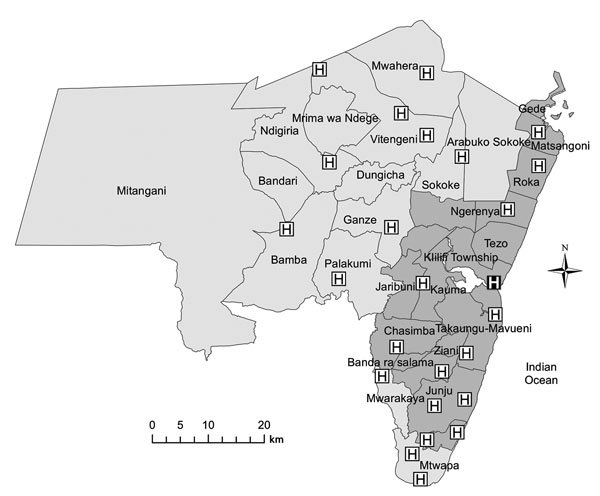
Kilifi District and the Kilifi Health and Demographic Surveillance Survey area (darker gray shading), showing administrative districts, Kilifi County Hospital (black square), and other tuberculosis treatment facilities (white squares), Kenya, 2010.

The study took place at Coast Provincial General Hospital (CPGH) and Kilifi County Hospital (KCH) in Coast Province, Kenya. CPGH provides primary and secondary care to the city of Mombasa and tertiary services for Coast Province. KCH is nested within the Kilifi Health and Demographic Surveillance System (KHDSS) (*13*), which covers a predominantly rural area of 891 km^2^ that in March 2011 was home to 261,919 residents in 29,970 households; two thirds of pediatric admissions to KCH during the study period were derived from this system. Three other health facilities in the KHDSS provide TB smear microscopy; 12 clinics are designated TB treatment centers (Figure 1). Because of resource constraints, contact tracing was not routine and isoniazid chemoprophylaxis not available at the time of the study, despite the inclusion of these steps in national TB guidelines.

### Participants

We established a system of enhanced passive and active childhood TB surveillance. In the passive case–detection arm, we prospectively recruited all children <15 years of age who were seen at KCH or CPGH during August 2009–July 2011 for an unexplained persistent cough for >2 weeks, pneumonia not responding to antibiotics, unexplained fever for >2 weeks, unexplained progressive weight loss or failure to thrive for >4 weeks, close contact with a person with TB, or clinical suspicion of TB for any other reason. Study clinicians and clinicians from the hospital and surrounding clinics were trained in the symptoms and signs of a range of TB presentations (Technical Appendix Table 1). We excluded children with an established alternative diagnosis that explained all the clinical features as well as children already on TB treatment for >2 weeks at presentation. In the active case–detection arm, we recruited KHDSS-resident children <5 years of age sharing a household with persons with new cases of smear-positive pulmonary TB.

### Clinical Procedures

All children underwent a similar structured history and examination, chest radiography, and tuberculin skin testing according to WHO guidelines (*14*) (online Technical Appendix). Children who were able to expectorate provided up to 3 spontaneous sputum samples. Sputum induction was performed on the remainder (*14*). Further investigations including extrapulmonary or repeat sputum sampling were performed at the discretion of the clinical team caring for the patient. Provider-initiated testing and counseling for HIV was performed according to national guidelines.

We classified children as having confirmed TB, highly probable TB, possible TB, or not TB (TB excluded) according to clinical, radiologic, and microbiological findings, based closely on stringent published definitions (Technical Appendix Table 2) (*15*,*16*). For comparison, we also applied other published clinical definitions to our dataset (Technical Appendix). Treatment protocols followed national guidelines. Children were followed up for 6 months or until a diagnosis of TB could be confidently excluded.

### Laboratory Methods

Acid-fast bacilli microscopy and mycobacterial culture using the BACTEC MGIT system (BD Diagnostics, Sparks, MD, USA) were performed according to standard protocols (*17*). Positive cultures were further characterized using the BD MGIT TBc Identification Test (BD Diagnostics) and Hain Genotype line probe assays (Hain Lifescience GmbH, Nehren, Germany), including isoniazid and rifampin drug-susceptibility testing. We performed the Xpert MTB/RIF assay version G4 (Cepheid, Sunnyvale, CA, USA) at the end of the study on specimens from all children treated for confirmed, highly probable, or possible TB as well as from children for whom a TB diagnosis had been excluded. Laboratory procedures were externally monitored using the United Kingdom National External Quality Assessment Service’s quality-assurance scheme (http://ukneqas.org.uk).

## Statistical Analysis

### Incidence Estimates

We used clinical data from KCH and event data from KHDSS to compile for every KHDSS-resident child a series of chronological time-span records representing the periods between consecutive birth, migration, enumeration, hospital presentation, or death events during the study period. We split these periods of observation by age category and estimated crude TB incidence rates as the total number of new TB cases identified (by both active and passive case detection) divided by the total person-years of observation in each age stratum. We compared estimates generated using the study case definitions with incidence estimates derived by applying other published clinical definitions of childhood TB to our dataset (Technical Appendix).

### Estimating the CDR

Crude incidence estimates assume all incident cases among KHDSS residents are captured by the study; however, hospital-based surveillance of childhood illnesses is known to be insensitive in this setting (*18*–*20*). We defined the CDR as the proportion of KHDSS-resident TB cases captured by the study. Because the actual number of children with TB is unknown, we used 3 different methods to estimate the CDR independently (detailed description in Technical Appendix).

#### TB Notification Data

We linked clinical data with National Tuberculosis Programme notification data and KHDSS census data. We estimated the CDR as 1) the proportion of KHDSS-resident smear-positive childhood TB cases reported to the National Tuberculosis Programme that were captured by passive case detection at KCH, and 2) the proportion of children’s household contacts of new smear-positive pulmonary TB cases captured by active contact tracing.

#### Hospital-Based Mortality Surveillance

We linked KHDSS vital status data with KCH admission data. We then calculated the proportion of all childhood deaths in the KHDSS area captured at KCH during the study period.

#### Verbal Autopsy

By using disease-specific mortality data from a contemporaneous verbal autopsy study of all deaths within the KHDSS (*21*), we estimated the proportion of childhood TB deaths captured by our study. Because the number of child TB cases diagnosed by verbal autopsy is small and healthcare-seeking behavior is usually determined by clinical features rather than diagnosis per se (*20*,*22*), we also estimated the CDR as the proportion of children who died having clinical features of suspected TB that were captured by the study.

To derive the most conservative estimates of the actual annual incidence of childhood TB, we divided crude incidence rates by the highest CDR estimate. We modeled the likely number of incident confirmed or highly probable TB (CHPTB) cases among children nationally by multiplying the total number of adult cases reported in Kenya in 2010 (*23*) by the ratio of child-to-adult cases in the KHDSS, assuming a similar ratio and adult CDR nationally. We then used denominator population data from the national census (*24*) to estimate the national incidence of childhood TB.

### Risk Factors for Childhood TB

We explored risk factors for childhood TB in a nested case–control analysis of children with CHPTB (cases) and children for whom TB was excluded (controls). To mitigate ascertainment bias in analysis of TB contact history, we excluded the small minority of children identified through active contact tracing. For each association, we derived crude odds ratios (ORs) and 95% CIs. We then included in a multivariable logistic regression model those variables with at least a weak association with TB in the univariable analysis (likelihood ratio test; p<0.1) and presented adjusted ORs and 95% CIs.

By using the number of KHDSS-resident adult cases reported to the National Tuberculosis Programme during the study period and the mean number of close contacts <5 years of age per case (*25*), we estimated the prevalence of household exposure to a person with confirmed TB among KHDSS-resident children <5 years of age. Using the contact status of CHPTB cases detected in the study, the child years at risk derived from the KHDSS census, and the exposure prevalence, we estimated the incidence of TB among contacts and noncontacts. The population attributable fraction for contact with a person with confirmed TB was calculated from the ensuing incidence rate ratio (IRR) and the exposure prevalence (p) by calculating p(IRR − 1)/1 + p(IRR − 1) (Technical Appendix).

## Results

**Figure 2:**
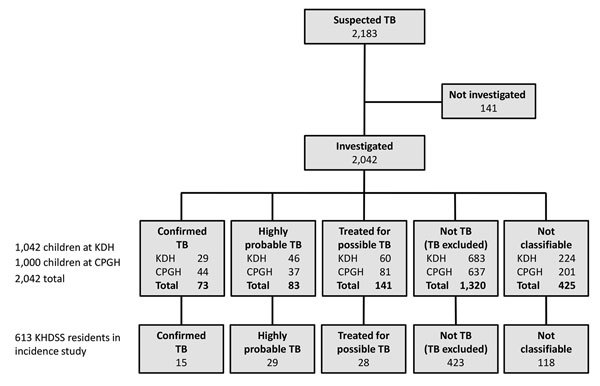
TB patient enrollment and disease classifications, Kilifi Health and Demographic Surveillance Survey, Kenya, August 2009–July 2011. A total of 141 children were not investigated (27 died, 40 were discharged, 3 were transferred, 1 self-discharged before workup completed, 30 defaulted outpatient follow-up, 40 had no reason documented). At KDH, 108/1,042 (10%) children investigated were identified through active contact tracing (2 confirmed TB, 4 highly probable TB, 87 not TB, 15 not classifiable). CPGH, Coast Provincial General Hospital; KDH, Kilifi District Hospital; KHDSS, Kilifi Health and Demographic Surveillance Survey; TB, tuberculosis.

We identified 2,183 children with suspected TB during the study period and summarized patient enrollment and diagnostic assignments (Figure 2). We excluded 141 (6%) children who died, were discharged, or were lost to follow-up before their diagnostic workups, including specimen collection for mycobacterial culture, could be completed (Figure 2). We summarized baseline clinical characteristics of the remaining 2,042 children included in the analyses (Table 1).

**Table 1:** Baseline characteristics of children with and without TB examined at Kilifi County Hospital and Coast Provincial General Hospital, Kenya, August 2009–July 2011*

Characteristic	Confirmed TB, n = 73		Highly probable TB, n = 83		Treated for possible TB, n = 141		Not TB/TB excluded, n = 1,320		Not classifiable, n = 425	
Case ascertainment										
Passive case detection	71 (97)		79 (95)		141 (100)		1,237 (94)		410 (96)	
Active case detection (contact tracing)	2 (3)		4 (5)		0 (0)		83 (6)		15 (4)	
Patient demographics										
Median age (interquartile range), mo	52 (16–114)		32 (13–70)		17 (10–64)		17 (10–41)		17 (9–44)	
0–4 y	38 (55)		59 (71)		99 (70)		1,119 (85)		345 (81)	
5–9 y	17 (25)		15 (18)		27 (19)		140 (11)		56 (13)	
10–14 y	18 (25)		9 (11)		15 (11)		61 (4)		24 (6)	
Sex										
M	39 (53)		43 (52)		70 (50)		696 (53)		224 (53)	
F	32 (47)		40 (48)		71 (50)		624 (47)		201 (47)	
Risk factors for TB										
HIV infected	17 (23)		21 (25)		42 (30)		160 (12)		112 (26)	
Severely malnourished	30 (41)		37 (45)		58 (41)		457 (35)		162 (38)	
BCG vaccination scar	65 (89)		86 (71)		128 (91)		1,172 (89)		338 (80)	
Close TB contact	36 (49)		33 (40)		27 (19)		246 (19)		78 (18)	
Clinical features of suspected TB										
Cough >2 wks	48 (66)		48 (58)		95 (67)		572 (43)		225 (53)	
Fever >2 wks	45 (62)		30 (36)		92 (65)		502 (38)		196 (46)	
Weight loss or failure to thrive >4 wks	42 (58)		39 (47)		77 (55)		575 (44)		208 (49)	
Pneumonia not responding to 1st-line ABX	27 (37)		25 (30)		42 (30)		308 (23)		159 (37)	
TB clinical syndrome										
Smear-positive pulmonary TB	20 (27)		4 (5)		0		–		–	
Smear-negative pulmonary TB	40 (55)		69 (83)		108 (77)		–		–	
All pulmonary TB†	60 (82)		73 (88)		108 (77)		–		–	
Extrapulmonary TB†	30 (41)		17 (20)		46 (33)		–		–	
Miliary TB	6 (8)		3 (4)		5 (4)		–		–	
TB meningitis	8 (11)		2 (2)		12 (9)		–		–	
Pleural TB	6 (9)		2 (2)		7 (5)		–		–	
TB lymphadenitis	6 (8)		6 (7)		6 (4)		–		–	
Osteoarticular TB	2 (3)		3 (4)		1 (1)		–		–	
Abdominal TB	9 (12)		2 (2)		10 (7)		–		–	
Persistent fever without a focus	0		1 (1)		13 (9)		–		–	
Drug resistance										
Isoniazid monoresistance	0		–		–		–		–	
Multidrug-resistant TB	1 (1.4)		–		–		–		–	


*Values are no. (%) unless otherwise indicated. ABX, antibiotics; BCG, bacillus Calmette-Guérin; TB, tuberculosis. †Some children had >1 focus of infection, including some with pulmonary TB and extrapulmonary TB. Among children with confirmed TB, microbiologic confirmation was required from >1 site; diagnosis of other sites of disease were based on the definitions of highly probable TB (online Technical Appendix Table 1, https://wwwnc.cdc.gov/EID/article/24/3/17-0785-Techapp1.pdf).

### Crude Incidence Estimates

We determined crude, hospital-based, age-specific incidence rates based on the study definitions (Table 2). The incidence of all childhood TB was 30.2 (95% CI 23.6–38.0) cases/100,000 children/year. The incidence of CHPTB was 18.4 (95% CI 13.4–24.7) cases/100,000 children/year; this estimate was very similar to that derived by retrospectively applying to our data consensus definitions of definite or probable TB that were published after completion of our study (*26*) (20.5 [95% CI 15.2–27.1]/100,000/year). Both figures are at the lower end of the range of estimates derived using published clinical definitions, which vary >30-fold (2.9–91.7/100,000/year) (Table 3).

**Table 2:** Crude hospital-based childhood TB incidence, by age group and diagnostic classification, Kilifi Health and Demographic Surveillance Survey, Kenya, August 2009–July 2011*

Characteristic	Confirmed TB, n = 73		Highly probable TB, n = 83		Treated for possible TB, n = 141		Not TB/TB excluded, n = 1,320		Not classifiable, n = 425	
Case ascertainment										
Passive case detection	71 (97)		79 (95)		141 (100)		1,237 (94)		410 (96)	
Active case detection (contact tracing)	2 (3)		4 (5)		0 (0)		83 (6)		15 (4)	
Patient demographics										
Median age (interquartile range), mo	52 (16–114)		32 (13–70)		17 (10–64)		17 (10–41)		17 (9–44)	
0–4 y	38 (55)		59 (71)		99 (70)		1,119 (85)		345 (81)	
5–9 y	17 (25)		15 (18)		27 (19)		140 (11)		56 (13)	
10–14 y	18 (25)		9 (11)		15 (11)		61 (4)		24 (6)	
Sex										
M	39 (53)		43 (52)		70 (50)		696 (53)		224 (53)	
F	32 (47)		40 (48)		71 (50)		624 (47)		201 (47)	
Risk factors for TB										
HIV infected	17 (23)		21 (25)		42 (30)		160 (12)		112 (26)	
Severely malnourished	30 (41)		37 (45)		58 (41)		457 (35)		162 (38)	
BCG vaccination scar	65 (89)		86 (71)		128 (91)		1,172 (89)		338 (80)	
Close TB contact	36 (49)		33 (40)		27 (19)		246 (19)		78 (18)	
Clinical features of suspected TB										
Cough >2 wks	48 (66)		48 (58)		95 (67)		572 (43)		225 (53)	
Fever >2 wks	45 (62)		30 (36)		92 (65)		502 (38)		196 (46)	
Weight loss or failure to thrive >4 wks	42 (58)		39 (47)		77 (55)		575 (44)		208 (49)	
Pneumonia not responding to 1st-line ABX	27 (37)		25 (30)		42 (30)		308 (23)		159 (37)	
TB clinical syndrome										
Smear-positive pulmonary TB	20 (27)		4 (5)		0		NA		NA	
Smear-negative pulmonary TB	40 (55)		69 (83)		108 (77)		NA		NA	
All pulmonary TB†	60 (82)		73 (88)		108 (77)		NA		NA	
Extrapulmonary TB†	30 (41)		17 (20)		46 (33)		NA		NA	
Miliary TB	6 (8)		3 (4)		5 (4)		NA		NA	
TB meningitis	8 (11)		2 (2)		12 (9)		NA		NA	
Pleural TB	6 (9)		2 (2)		7 (5)		NA		NA	
TB lymphadenitis	6 (8)		6 (7)		6 (4)		NA		NA	
Osteoarticular TB	2 (3)		3 (4)		1 (1)		NA		NA	
Abdominal TB	9 (12)		2 (2)		10 (7)		NA		NA	
Persistent fever without a focus	0		1 (1)		13 (9)		NA		NA	
Drug resistance										
Isoniazid monoresistance	0		NA		NA		NA		NA	
Multidrug-resistant TB	1 (1.4)		NA		NA		NA		NA	


*Values are no. (%) unless otherwise indicated. ABX, antibiotics; BCG, bacillus Calmette-Guérin; NA, not applicable; TB, tuberculosis. †Some children had >1 focus of infection, including some with pulmonary TB and extrapulmonary TB. Among children with confirmed TB, microbiologic confirmation was required from >1 site; diagnosis of other sites of disease was based on the definitions of highly probable TB (online Technical Appendix Table 1, https://wwwnc.cdc.gov/EID/article/24/3/17-0785-Techapp1.pdf).

**Table 3:** Incidence of childhood TB derived by applying other published clinical definitions, algorithms, and guidelines, in order of increasing incidence, Kilifi Health and Demographic Surveillance Survey, Kenya, August 2009–July 2011*

Author, year (reference)	Outcomes defined	No. cases	Incidence, cases/100,000 children/y (95% CI)†
WHO, 2006 (*27*)	(a) Strongly suggestive of TB‡	7	2.9 (1.2–6.0)
Stegen (*28*)	(a) Probable TB	18	7.5 (4.5–11.9)
Nair (*29*)	(a) “TB appears unquestionable”	28	11.7 (7.8–17.0)
WHO, 2006 (*27*)	(b) Requires investigation for TB‡	33	13.8 (9.5–19.4)
Graham (*26*)	Probable TB	42	17.6 (12.7–23.8)
Hawkridge (*30*)	Probable TB	54	22.6 (17.0–29.5)
Nair (*29*)	(b) TB probable or “unquestionable”	55	23.0 (17.4–30.0)
Stoltz (*31*)	Probable TB	73	30.6 (24.0–38.5)
Jeena (*32*)	Probable TB	107	44.8 (36.7–54.2)
Edwards (*33*)	Criteria for TB treatment	110	46.1 (37.9–55.5)
Ghidey (*34*)	(a) Criteria for TB treatment§	113	47.3 (39.0–56.9)
WHO, 1983 (*35*)	Probable TB	116	48.6 (40.2–58.3)
Ramachandran (*36*)	Criteria for TB treatment	118	49.4 (40.9–59.2)
Ghidey (*34*)	(b) Criteria for TB treatment§	130	54.5 (45.4–64.7)
Stegen (*28*)	(b) Probable or possible TB	136	57.0 (47.8–67.4)
Graham (*26*)	Probable or possible TB	145	60.7 (51.3–71.5)
Osborne (*37*)	Probable TB	159	66.6 (56.7–77.8)
Fourie (*38*)	High probability of TB¶	162	67.9 (57.8–79.2)
Cundall (*39*)	Probable TB	207	86.7 (75.3–99.4)
Kiwanuka (*40*)	Probable TB	219	91.7 (80.0–104.7)

*TB, tuberculosis; WHO, World Health Organization. †Denominator for incidence calculations is the total person-years observation among children age <15 y (N = 238,746). ‡Results shown separately for (a) children whose clinical features “strongly suggest a diagnosis of TB” according to the guidelines, and (b) using broader criteria that included under “physical signs highly suggestive of TB” all the other “suggestive clinical signs” listed as requiring investigation for TB. §Results for Ghidey and Habte tool (*34*) shown using both (a) >3 and (b) >2 signs and symptoms to define a “suggestive symptom complex of TB” (online Technical Appendix Table 1, https://wwwnc.cdc.gov/EID/article/24/3/17-0785-Techapp1.pdf). ¶For the purposes of our analyses, we used “score 2” proposed by Fourie et al (*38*), which was derived in high TB burden settings in South Africa, Madagascar, and Nicaragua.

### CDR and Adjusted Incidence Estimates

CDR estimates derived using TB notifications, KHDSS census data, and verbal autopsy ranged from 0.2 to 0.35 (Table 4), substantially lower than the estimated CDR of 0.82 for adults in Kenya (*41*). Hospital-based mortality surveillance provided the largest and most precise estimate of the CDR (0.35 [95% CI 0.31–0.40]), so we used this to derive the most conservative estimates of the actual community incidence of childhood TB (Table 5). After adjustment for CDR, the incidence of CHPTB and all TB among children in the KHDSS was 53 (95% CI 38–71) and 86 (95% CI 67–109) cases/100,000/year, respectively.

**Table 4:** Case detection rate estimates derived by using TB notifications, Kilifi Health and Demographic Surveillance Survey census data, and verbal autopsy methods, Kenya, August 2009–July 2011*

Method	Calculation of CDR estimate	CDR estimate (95% CI)
TB notifications		
Passive case detection		0.30 (0.07–0.65)
Active contact tracing		0.20 (0.13–0.26)
KHDSS census		
Mortality surveillance		0.35 (0.31–0.40)
Verbal autopsy		
TB deaths		0.20 (0.03– 0.56)
TB suspected deaths		0.22 (0.15–0.32)

*CDR, case detection rate; KCH, Kilifi County Hospital; KHDSS, Kilifi Health and Demographic Surveillance Survey; TB, tuberculosis, VA, verbal autopsy.

**Table 5:** Estimated annual caseload and incidence of childhood TB after adjustment for the case detection rate, Kilifi Health and Demographic Surveillance Survey, August 2009–July 2011*

TB classification	Age group, y	No. cases	Adjusted incidence, cases/100,000 children/y (95% CI)
Confirmed TB	0–4	20	22 (9–46)
	5–9	17	22 (8–47)
	10–14	6	9 (1–29)
	Total	43	18 (10–30)
Confirmed or highly probable TB	0–4	86	96 (65–137)
	5–9	31	39 (20–71)
	10–14	9	13 (3–36)
	Total	126	53 (38–71)
All TB	0–4	131	146 (107–196)
	5–9	60	76 (47–116)
	10–14	14	20 (7–48)
	Total	205	86 (67–109)

*To generate the most conservative estimates of community childhood TB incidence, we used the highest case detection rate estimate of 0.35 derived from hospital-based mortality surveillance. TB, tuberculosis.

### Implications for the National Incidence of Childhood TB

During August 2009–July 2011, a total of 678 new cases of adult TB were reported to the National Tuberculosis Programme, and an estimated 126 new CHPTB cases were reported in children (Table 5) among KHDSS residents. Nationally 89,883 adult and 5,721 child TB cases were reported in 2010 (*41*) among a population that includes ≈17.6 million children <15 years of age (*24*). Applying the ratio of adult-to-child TB cases in the KHDSS to the national caseload yields an estimated 16,704 new CHPTB cases among children <15 years of age nationally in 2010, suggesting a national childhood TB CDR of 29% and incidence of 95 cases/100,000 children/year (Technical Appendix Table 3).

### Risk Factors for Childhood TB

We summarized associations of CHPTB and important putative risk factors (Table 6). A history of known close TB contact at presentation was strongly associated with CHPTB, with an effect gradient according to the contacts’ smear status, proximity, relationship, and number (Technical Appendix Table 4). No child case-patients with a close TB contact had received isoniazid chemoprophylaxis. We observed a weaker association with HIV and in young children with severe malnutrition but no association between the presence of a bacillus Calmette-Guérin (BCG) vaccination scar and TB, although power to detect an effect was low because of the small proportion of children without a BCG vaccination scar.

**Table 6:** Crude and adjusted odds ratios for risk factors associated with confirmed or highly probable TB among children examined at Kilifi County Hospital and Coast Provincial General Hospital, Kenya, August 2009–July 2011*

Age group	Cases			Controls		Crude OR for TB (95% CI)	p value		aOR for TB (95% CI)	p value	
	Factor present	Factor absent		Factor present	Factor absent						
Children <5 y											
HIV infection†	17	73		112	872	1.8 (1.0–3.2)	0.036		1.3 (0.7–2.4)	0.321	
Severe malnutrition‡	56	35		413	620	2.4 (1.5–3.7)	<0.001		2.6 (1.6–4.1)	<0.001	
BCG vaccination scar	82	9		921	112	1.1 (0.5–2.3)	0.779		–		
Close TB contact	33	58		125	908	4.1 (2.6–6.6)	<0.001		5.1 (3.1–8.3)	<0.001	
Children 5–14 y											
HIV infection†	21	38		47	143	1.7 (0.9–3.2)	0.103		1.5 (0.8–2.9)	0.229	
Severe malnutrition‡	9	50		43	157	0.7 (0.3–1.4)	0.294		–		
BCG vaccination scar	48	11		173	27	0.7 (0.3–1.5)	0.327		–		
Close TB contact	30	29		34	166	5.1 (2.6–9.9)	<0.001		5.2 (2.7–9.8)	<0.001	
All children <15 y											
HIV infection†	38	111		159	1,015	2.2 (1.5–3.3)	<0.001		1.9 (1.2–2.9)	0.003	
Severe malnutrition‡	65	85		456	777	1.3 (0.9–1.8)	0.130		–		
BCG vaccination scar	130	20		1,094	139	0.8 (0.5–1.4)	0.455		–		
Close TB contact	63	87		159	1,074	5.0 (3.4–7.3)	<0.001		5.0 (3.4–7.2)	<0.001	

*aOR, adjusted odds ratio; BCG, bacillus Calmette-Guérin; OR, odds ratio; TB, tuberculosis. †HIV status was missing for 1/150 (0.7%) cases and 59/1233 (4.8%) controls. ‡Severe malnutrition defined according to World Health Organization guidelines as weight-for-age z-score of <3 or the presence of nutritional edema (*42*).

### Preventable TB Burden among Child Household TB Contacts

Among KHDSS-resident children <5 years of age, an estimated 1,259 were close contacts of adults with new TB cases reported during the study period. The incidence of CHPTB was 596 cases/100,000/year among children with a close TB contact and 17 cases/100,000/year among those without a close TB contact, yielding a 49% population attributable fraction for having a recent and known TB contact (Technical Appendix Table 5).

## Discussion

This study provides rare prospective empiric data on the TB incidence and CDR among children <15 years of age in Kenya, a country with a high TB burden, and is one of few prospective incidence studies globally (*3*). This community-based study was nested in a demographic surveillance survey, underpinned by enhanced active and passive surveillance, mycobacterial culture facilities, and linked hospital, demographic, notification, and verbal autopsy data. We used a hierarchical diagnostic classification in keeping with recommendations for childhood TB surveillance and research (*26*,*35*). A comprehensive algorithm of clinical, radiologic, and laboratory investigations combined with careful follow-up of children enrolled in the KIDS TB Study ensured diagnostic classifications were optimized within the limitations of currently available diagnostic tools.

Although the diagnosis of confirmed TB has the highest specificity, the poor sensitivity of mycobacterial culture for childhood TB diagnosis means that incidence estimates based only on confirmed cases will underestimate the actual disease burden. Conversely, including possible TB cases in the numerator might overestimate incidence. Most children in the highly probable TB group probably did have TB, given the stringent diagnostic criteria, and although the sensitivity of this classification is not perfect, it probably captured many of the actual cases of active TB for which culture confirmation was not obtained. We therefore used a combination of confirmed or highly probable TB (CHPTB) as the measure most likely to optimize sensitivity and specificity for estimation of childhood TB incidence.

Compared with estimates based on published clinical definitions, our measure of CHPTB incidence is among the most conservative, similar to the estimate obtained by retrospectively applying more recent consensus definitions for research (*26*). Even after inclusion of all TB cases, our measure remained among the lowest, suggesting that many published clinical definitions would overdiagnose TB in this and similar settings were they to be applied routinely in clinical practice. The huge range in incidence estimates derived using different case definitions emphasizes the difficulty in interpreting existing disease burden data and the need for high-quality prospective incidence studies to improve disease burden estimates.

Robust community incidence estimates depend on high-quality diagnosis to minimize misclassification as well as a high CDR. Broad screening criteria for all children admitted to hospital with any features of suspected TB, plus active case detection through contact tracing, ensured that case ascertainment at KCH was optimized. Nevertheless, the social, financial, and geographic barriers to obtaining hospital care in this setting mean that many ill KHDSS-resident children are not seen at KCH (*18*–*20*). Furthermore, challenges in childhood TB diagnosis, combined with limited diagnostic resources, make surveillance data from other health facilities unreliable. We therefore estimated the CDR of hospital-based surveillance at KCH by using 3 independent techniques. Each measure is necessarily a surrogate, and each has limitations, but the similarity of these estimates supports their validity.

Because we used the highest CDR estimate to generate conservative estimates of childhood TB incidence, the projected national incidence was 3 times higher than that reported. Nevertheless, the projected ratio of adult-to-child TB cases is still consistent with other studies in Africa (*43*,*44*) and with recent global estimates (*1*,*5*,*6*,*9*), although lower than some regional and global figures (*3*). Other estimates of the global TB burden have indicated a lower proportion of childhood cases (*7*,*8*). However, in the absence of data from children, those estimates assume a similar CDR for adults (*8*) or impute missing data based on reported proportions of smear-negative and extrapulmonary TB by age group (*7*), assumptions that have been challenged (*11*,*45*). Our study provides important empirical data on the probable CDR among children. The results suggest that the CDR among children is substantially lower than among adults and support estimates derived using other modeling approaches (*5*,*6*), including recently revised WHO estimates of global childhood TB incidence that assume a CDR of 36% (*9*).

The strong association of childhood TB with a history of close TB contact has 2 important implications for clinical practice and public health policy. First, eliciting a history of TB contact should be a standard part of the assessment of every ill child in TB-endemic settings. Among inpatients in our study, 1 in 5 with a known close TB contact had CHPTB. Early identification and investigation of this high-risk group might improve clinical outcomes through earlier diagnosis and treatment.

Second, and most important, our finding that 49% TB cases among children <5 years of age were attributable to a known household TB contact suggests that half the CHPTB cases in young children might have been prevented by chemoprophylaxis. Estimating the population attributable fraction of contact with a person with confirmed TB provides a novel approach for assessing the potential impact of TB chemoprophylaxis at the population level that might be applied to other settings. Our results from Kenya support recent global estimates of TB burden among child TB contacts (*25*). By demonstrating a large potential impact on childhood TB incidence, our findings provide further strong endorsement for existing policy recommendations for TB chemoprophylaxis (*25*,*46*).

Extrapolation of results from a single district must be interpreted with caution. Childhood TB incidence and the contribution of childhood TB cases to the total TB burden are likely to be affected by factors that vary geographically, including community TB prevalence; social and demographic factors, such as urbanization, that affect the annual risk for TB infection; prevalence of host factors, such as BCG vaccination, HIV infection, and malnutrition; and local population structures. Therefore, we did not attempt simply to age-standardize the Kilifi incidence rates to the national population of children in Kenya.

We reasoned instead that the proportion of the total TB caseload accounted for by children is probably less prone to geographic variation, and estimated the national burden of childhood TB by assuming that the CDR among adults and the ratio of adult-to-child cases is the same in the KHDSS and nationally. Importantly, the age structures of the KHDSS and Kenya are very similar (*13*,*24*), suggesting that age is unlikely to confound this approach. Compared with Kilifi, the higher estimate of TB incidence nationally is consistent with greater urbanization (*13*,*24*) and a higher annual risk for TB infection (*47*), HIV prevalence (*24*), and overall TB incidence (*1*). Because ecologic data suggest that the pediatric proportion of cases actually increases with increasing overall TB incidence (*6*,*12*), this approach might underestimate the actual national childhood TB burden. Our restriction of TB cases to those that met the stringent criteria of CHPTB and our adjustment of hospital-based incidence rates using the highest CDR estimate also suggest that our estimates are conservative.

In conclusion, by using a combination of clinical, laboratory, and epidemiologic resources not usually available for routine surveillance, we have estimated the incidence of childhood TB in Kenya. Although this study is very resource-intensive, the wide range of incidence estimates based on existing clinical definitions highlights the difficulty in interpreting routine notification data and reinforces the need for similar studies in a range of different epidemiologic settings. In a setting where routine facilities for childhood TB diagnosis are typical of most countries with a high TB burden, our results also provide important empirical data on the TB CDR among children. The results support recently improved WHO estimates of global childhood TB incidence based on modeling approaches, which assume a very similar CDR (*1*,*9*). Our findings also reinforce the urgent need to improve case detection among children to reduce childhood TB mortality (*48*). Crucially, they suggest that half the TB cases in young children might be prevented by implementing existing WHO guidelines for contact tracing and chemoprophylaxis.

Technical Appendix Description of methods and supplementary results of the Kilifi Health and Demographic Surveillance Survey, Kenya, August 2009–July 2011.
