# Supplementary material for: Prospective Observational Study of Incidence and Preventable Burden of Childhood Tuberculosis, Kenya
Source: Emerg Infect Dis. 2018 Mar;24(3):514–23. doi: 10.3201/eid2403.170785 (PMC5823335; doi:10.3201/eid2403.170785)
Supplement: Technical Appendix — Description of methods and supplementary results of the Kilifi Health and Demographic Surveillance Survey, Kenya, August 2009–July 2011. [file 17-0785-Techapp-s1.pdf]

Article DOI: https://doi.org/10.3201/eid2403.170785

Prospective Observational Study of Incidence and Preventable Burden of Childhood Tuberculosis, Kenya

Technical Appendix

Investigation of Children Enrolled through Passive Case Detection at KCH

All children who presented to KCH with clinical features of suspected TB were weighed and underwent a structured history and examination, including questions about any history of known TB contact at the time of presentation. Tuberculin skin testing (TST) was performed using the Mantoux method and 2 tuberculin units (TU) of tuberculin PPD RT23 (*1*). A positive TST was defined as a diameter of induration ≥10mm, or ≥5mm in HIV infected or severely malnourished children (*1*). Children admitted to KCH also had a full blood count (FBC; Beckman/Coulter, Fullerton, UK), thick and thin blood films for malaria parasites (malaria parasite slide, MPS), and a blood culture (BACTEC PedsPlus, Becton Dickinson, CA, USA). Provider initiated testing and counseling (PiTC) for HIV was performed according to Kenyan national guidelines, which recommend testing for all inpatients and for all patients investigated for TB, on an opt-out basis (*2*,*3*).

All children had a posterior-anterior (PA) or anterior-posterior (AP) chest x-ray (CXR). Lateral CXRs were performed at the discretion of the clinician after reviewing the PA/AP CXR, for example to assess further any suspected hilar lymphadenopathy. CXRs were read independently by the study clinician (AJB) and by a pediatric radiologist (JS) blinded to the clinical details. Data were entered onto a standardized reporting form closely based on consensus guidelines (*4*). Discrepancies between these two readers were resolved by a second pediatric radiologist (KP) also blinded to both the clinical details and the findings of the first two readers.

Other blood tests and more specialist investigations (e.g., imaging) were performed at the discretion of the clinical team caring for the child.

Investigation of Children Enrolled through Active Contact Tracing

New cases of smear positive pulmonary TB resident within the Kilifi Health and Demographic Surveillance System (KHDSS) were identified in the KCH TB outpatient clinic, and child household contacts of these index cases identified on the KHDSS population register. For pragmatic reasons, and in keeping with Kenyan national guidelines, contact tracing focused on children under 5 years of age resident in the same household as a case of smear positive pulmonary TB, as smear positive cases are the most infectious and young children are most vulnerable to developing active TB following infection (*5*). Each index case was then invited to bring all children under 5 years in the household (symptomatic or asymptomatic) to the pediatric TB outpatient clinic for further assessment, and given sufficient money to cover the return fare to hospital.

All children identified through active contact tracing underwent a structured history and examination, anthropometry, CXR, and a TST. Those with symptoms or signs of possible TB (Technical Appendix Box 2), an abnormal CXR, or a positive TST were further investigated for suspected TB as described below.

Specimen Collection for Mycobacteriology

Appropriate clinical specimens were collected for AFB microscopy and mycobacterial culture from all children with suspected TB. Children who were able to expectorate provided three spontaneous sputum samples. Sputum induction was performed on the remainder. If sputum induction was contraindicated (e.g., due to severe respiratory distress), gastric aspiration was performed. Sputum induction and gastric aspiration were performed according to international recommendations (*6*). Further investigations including fine needle aspiration (FNA) of lymph nodes, mycobacterial culture of CSF, urine, pleural/ascitic/joint fluid, or biopsy material, or repeat sampling, were performed at the discretion of the clinical team caring for the patient according to clinical indications in individual cases. Specimens were transported to the laboratory at 2–8°C and processed the same day.

Supplementary Statistical Methods

Application of Published Clinical Diagnostic Tools to Estimate Childhood TB Incidence

To compare crude incidence estimates generated using the study case definitions with incidence estimates derived using other published clinical definitions we included clinical diagnostic tools published in the peer reviewed medical literature, and guidelines from the WHO and Kenya National TB Programme. We excluded published tools that failed to present diagnostic criteria in sufficient detail to apply them to the dataset. For those tools that included a category of confirmed TB based on microbiological diagnosis we confined our analysis to categories defined by clinical criteria alone, to explore their performance under normal programmatic conditions with limited availability of mycobacterial culture. We also retrospectively applied new consensus definitions for childhood TB research that were published after completion of our study (*4*), and derived incidence estimates for the consensus definitions of both Definite and Probable TB to facilitate comparison with future studies.

We created variables for each diagnostic score and/or diagnostic categories with close reference to the published definitions of each variable. In instances where the exact definition of a clinical variable was not clearly specified in the original publication we chose what we judged to be the most likely intended definition for application in the relevant setting and reported the definition we used. Thus 'unexplained fever' was defined as a fever for >14 days in the absence of malaria parasitaemia or evidence of focal infection; a cutoff of at least 1 week's duration was used for a history of night sweats; and 'bulky lymphadenopathy' was defined pragmatically as the presence of lymph nodes sufficiently large to perform a fine needle aspirate (usually ≥2cm diameter). 'Malnutrition not responding to treatment' was defined as death, or failure to regain 10% bodyweight (in the case of marasmus) or failure of edema to resolve (kwashiorkor), in a child admitted with severe malnutrition.

A 'suggestive symptom complex of TB' was included in the Ghidey-Habte diagnostic tool (*7*) but only vaguely defined as "*non-specific symptoms such as fever, night sweats and loss of weight, and specific symptoms related to the site invaded, e.g. cough, swelling of lymph nodes, abdominal distension, difficulty in walking, etc* ". For the purposes of our analysis we included in this definition fever, cough, night sweats, and weight loss (each for at least 2 weeks), bulky lymphadenopathy, signs of pleural effusion or ascites, gibbus, and a change in temperament or

reduced level of consciousness. We then compared incidence estimates using a requirement for either ≥2 or ≥3 of these clinical features to define a 'suggestive symptom complex of TB'.

In keeping with published definitions (*4,8,9*), a 'suggestive CXR' for TB was defined as the presence of a Ghon focus or complex, miliary infiltrate, cavities, or a pleural or pericardial effusion - unless an alternative definition was clearly presented for a particular clinical tool in which case the definition presented was used.

Using each of these published clinical definitions we calculated TB incidence using as the numerator the number of KHDSS-resident children fulfilling each definition during the study period.

Estimating the Case Detection Rate

Using TB Notification Data

We linked National Tuberculosis Programme (NTP) notification data with KHDSS census data to estimate the CDR in both the passive and the active case detection arms of the study.

Passive Case Detection

We used notification data from the Kilifi District TB Register to estimate the proportions of KHDSS resident child TB cases captured at KCH through passive case detection. Data from the register were double entered into a bespoke electronic database using Filemaker Pro version 10 (Filemaker Inc, CA, USA). The KHDSS residence status (resident or non-resident) of each patient in the register was then coded manually by a senior demographer with several years of local experience and detailed knowledge of the KHDSS area (CN), using the address documented in the register. All KHDSS resident childhood TB cases notified between August 2009 and July 2011 were identified from this database. We then manually cross-referenced the name, age and treatment date of each of these cases against the KIDS TB Study database to identify children that had also been captured by passive case detection at KCH. To limit disease misclassification among young children we limited the analysis to smear positive cases, and calculated the case detection rate as:

$$CDR = \frac{\text{No. KHDSS resident, smear-positive child TB cases captured by the KIDS TB Study}}{\text{Total no. KHDSS resident smear-positive child TB cases}}$$

Active Case Detection

Case ascertainment of children aged 0 to 4 years in the active (contact tracing) case detection arm depended first on identification of all KHDSS resident cases of smear positive pulmonary TB; and second on each of these smear positive index cases bringing their child household contacts to the pediatric TB clinic for investigation. By linking smear and residence data from the Kilifi District TB Register with data from our register of all smear positive pulmonary TB patients seen in the KCH TB clinic we determined the proportion of all notified smear positive pulmonary TB cases from the KHDSS area that were captured at KCH. We identified from the KHDSS census the number of child household contacts under 5 years old for each index case, and thereby the total number of eligible child household contacts under 5 years old and resident in the KHDSS area. We assumed that the average number of child household contacts was similar among index cases who presented to KCH and elsewhere, and that the risk of TB among contacts was independent of where the index case presented or whether the child was brought to the pediatric TB clinic for investigation. The case detection rate in the active case detection arm was then calculated as:

$$CDR = \frac{\text{No. index cases captured at KCH}}{\text{Total no. index cases}} \times \frac{\text{No. eligible child contacts investigated}}{\text{Total no. eligible child contacts identified at KCH}}$$

We derived 95% confidence intervals based on the variance of the product of these two proportions using standard methods.

Using Hospital-Based Mortality Surveillance

We used the unique personal identification number (PID) of each KHDSS resident child to link vital status data from KHDSS census rounds with KCH pediatric admission outcome data. We then calculated the case detection rate as the proportion of all childhood deaths in the KHDSS area that were captured at KCH during the study period:

$$CDR = \frac{\text{No. KHDSS-resident children who died at KCH}}{\text{Total no. childhood deaths among KHDSS residents}}$$

Using Verbal Autopsy

A better approach to estimating the sensitivity of hospital-based surveillance is to use *disease specific* mortality data to calculate the proportion of childhood TB deaths captured by the study. Poor quality vital registration data in Kilifi District make these data unsuitable for this

analysis. We therefore made use of data from an ongoing verbal autopsy (VA) study within the KHDSS.

Details of the Kilifi verbal autopsy study, including validation of the methodology using hospital records of the cause of death, have been published elsewhere (*10*). Deaths among KHDSS residents are identified by the thrice yearly enumeration rounds, and relatives of the deceased are then visited at home as soon as possible after the locally accepted 1 month bereavement period. Following consent, verbal autopsy is performed using the WHO Sample Vital Registration with Verbal Autopsy (SAVVY) tool. Structured questionnaires include an initial narrative section with open questions about the circumstances of death, followed by a series of closed questions that provide detailed information about the medical history and associated clinical features. Two independent clinicians then code the causes of death in each case according to a standard rubric and the WHO International Statistical Classification of Diseases Version 10 (ICD10). In the case of a discrepancy between the two clinicians, a third clinician reviews the case blind to adjudicate, and if there is no agreement between the three reviewers they meet to discuss the case to form a consensus.

Using each child’s unique KHDSS personal identification (PID) number we merged VA and KIDS TB records to calculate the proportion of TB deaths among KHDSS resident children with that were captured by the KIDS TB study. We defined TB deaths as those whose cause was coded as TB or which occurred in a patient with documented tuberculosis according to the respondent and/or any available supporting documentation, including death certificates, burial permits and post mortem reports.

We then estimated the case detection rate as

$$CDR = \frac{\text{No. TB deaths in VA study that occurred in children captured by KIDS TB Study}}{\text{No. TB deaths in VA study}}$$

Although the true mortality burden of TB among children in Kilifi District was not known, we predicted that the number of child TB cases diagnosed by VA was likely to be small (since TB is responsible for a minority of childhood deaths and is even more difficult to diagnose retrospectively by VA than in clinical practice); and that the precision of our case detection rate estimate was therefore likely to be poor.

To mitigate this, we also used the VA study to identify the much larger group of children whose reported clinical features before death met the KIDS TB Study criteria for suspected TB. Healthcare-seeking behavior in Kilifi is usually determined by the clinical features of an illness, rather than the diagnosis per se (*11*,*12*). We reasoned, therefore, that of all children with clinical features of suspected TB who died, the proportion captured by the KIDS TB Study would provide a surrogate measure of the case detection rate:

$$CDR = \frac{\text{No. TB suspect deaths in VA study that were captured by KIDS TB Study}}{\text{No. TB suspect deaths in VA study}}$$

For the purposes of this analysis we defined '*pneumonia not responding to first line antibiotics*' as death due to pneumonia despite reported treatment.

Risk Factors for Childhood TB

We summarized the distribution among cases and controls of each putative risk factor, and derived crude odds ratios (OR) and 95% confidence intervals (CI) in each case. Likelihood ratio tests for a general association were performed and p values reported.

To explore associations with TB contact variables we used children with no TB contact as the baseline group for comparison. Since some children had a history of more than one TB contact, we assumed an individual child had an equal probability of acquiring TB from each contact; created a separate record for each child-contact pair; and weighted each of these pairs in the analysis by the reciprocal of the number of TB contacts reported for each child.

We then derived multivariable logistic regression models to identify independent risk factors for TB. Categorical variables with at least a weak association with TB in the univariable analysis (likelihood ratio test p value ≤0.1) were included in the model. We performed backward stepwise logistic regression using standard selection criteria, such that variables that were not significantly associated with TB (Wald p value <0.5) were sequentially dropped from the model. Likelihood ratio tests were used to test for potential interactions in the final model. Based on this model adjusted odds ratios and 95% confidence intervals were derived for the associations with TB of each variable included; p values for each association were derived using the Wald test.

We estimated the population attributable fraction (PAF) of childhood TB due to close contact with a known case of adult TB. We confined this analysis to children under 5 years for two reasons. First, it is well documented from natural history studies in the pre-chemotherapy era that >90% active TB disease in this age group occurs within 2 years of infection (*13*). The number of contacts identified during the 2 year recruitment period therefore provides a good estimate of the likely number of contacts putting children at risk, since the overall rate of TB notifications in the study population is constant. Second, this is the group targeted for isoniazid chemoprophylaxis since they are the most vulnerable (*1*).

We multiplied the number of notified KHDSS-resident TB cases among adults (TB_{khdss}) by the mean number of child household contacts under 5 years old per TB case ($\bar{C}_{household}$) to estimate the number of KHDSS-resident children with a known household TB contact ($N_{contacts}$) during this study period:

$$N_{contacts} = TB_{khdss} \times \bar{C}_{household}.$$

We then estimated the number of person years observation among children <5 years old with a known household TB contact ($pyo_{contacts}$) as

$$pyo_{contacts} = N_{contacts} \times 2years.$$

We calculated the incidence of TB among children <5 years old with and without a history of household TB contact as

$$I_{contacts} = \frac{tb_{contacts}}{pyo_{contacts}} \text{ and } I_{non-contacts} = \frac{tb_{non-contacts}}{pyo_{total} - pyo_{contacts}},$$

where $tb_{contacts}$ and $tb_{non-contacts}$ are the numbers of TB cases among children <5 years old with and without a known history of TB contact, and pyo_{khdss} is the total person years observation among children <5 years old resident in the KHDSS. The Incident Rate Ratio (*IRR*) was then calculated as

$$IRR = \frac{I_{contacts}}{I_{non-contacts}}$$

The community prevalence of household TB contact (*p*) among KHDSS-resident children <5 years old during the 2 year study period was calculated as

$$p = \frac{N_{contacts}}{N_{total}} = \frac{pyo_{contacts}}{pyo_{total}}$$

Finally we calculated the PAF for contact with a notified adult case of TB as

$$PAF = \frac{p(IRR - 1)}{p(IRR - 1) + 1}$$

References

1. World Health Organization. Guidance for national tuberculosis programmes on the management of tuberculosis in children. Geneva: World Health Organization; 2006.

2. Kenya Ministry of Public Health and Sanitation. National guidelines for HIV testing and counselling in Kenya. In: National AIDS & STI Control Programme. 2nd edition. Nairobi, Kenya: Kenya Ministry of Public Health and Sanitation; 2010.

3. Division of Leprosy Tuberculosis and Lung Diseases. Division of Leprosy Tuberculosis and Lung Diseases guidelines. Nairobi: Kenya Ministry of Public Health and Sanitation; 2008.

4. Graham SM, Ahmed T, Amanullah F, Browning R, Cardenas V, Casenghi M, et al. Evaluation of tuberculosis diagnostics in children: 1. Proposed clinical case definitions for classification of intrathoracic tuberculosis disease. Consensus from an expert panel. J Infect Dis. 2012;205(Suppl 2):S199–208. PubMed http://dx.doi.org/10.1093/infdis/jis008

5. Newton SM, Brent AJ, Anderson S, Whittaker E, Kampmann B. Paediatric tuberculosis. Lancet Infect Dis. 2008;8:498–510. PubMed http://dx.doi.org/10.1016/S1473-3099(08)70182-8

6. World Health Organization. Guidance for national tuberculosis programmes on the management of tuberculosis in children. Second edition. Geneva: World Health Organization; 2014.

7. Ghidey Y, Habte D. Tuberculosis in childhood: an analysis of 412 cases. Ethiop Med J. 1983;21:161–7. PubMed

8. Gie RP. Diagnostic atlas of intrathoracic tuberculosis in children. A guide for low-income countries. Paris: International Union Against Tuberculosis and Lung Disease; 2003.

9. Marais BJ, Gie RP, Schaaf HS, Starke JR, Hesseling AC, Donald PR, et al. A proposed radiological classification of childhood intra-thoracic tuberculosis. Pediatr Radiol. 2004;34:886–94. PubMed http://dx.doi.org/10.1007/s00247-004-1238-0

10. Bauni E, Ndila C, Mochamah G, Nyutu G, Matata L, Ondieki C, et al. Validating physician-certified verbal autopsy and probabilistic modeling (InterVA) approaches to verbal autopsy interpretation using hospital causes of adult deaths. Popul Health Metr. 2011;9:49. PubMed http://dx.doi.org/10.1186/1478-7954-9-49

11. Molyneux CS, Mung'Ala-Odera V, Harpham T, Snow RW. Maternal responses to childhood fevers: a comparison of rural and urban residents in coastal Kenya. Trop Med Int Health. 1999;4:836–45. PubMed http://dx.doi.org/10.1046/j.1365-3156.1999.00489.x

12. Molyneux CS, Murira G, Masha J, Snow RW. Intra-household relations and treatment decision-making for childhood illness: a Kenyan case study. J Biosoc Sci. 2002;34:109–31. PubMed

13. Marais BJ, Gie RP, Schaaf HS, Hesseling AC, Obihara CC, Starke JJ, et al. The natural history of childhood intra-thoracic tuberculosis: a critical review of literature from the pre-chemotherapy era. Int J Tuberc Lung Dis. 2004;8:392–402. PubMed

14. Kenya National Bureau of Statistics. Kenya Demographic and Health Survey 2008–09. Nairobi, Kenya: Kenya National Bureau of Statistics; 2010.

Technical Appendix Table 1. Symptoms and signs of possible TB

Symptoms of possible TB	Signs of possible TB
Any of the following:<br>• fever, night sweats, weight loss, lethargy, or failure to thrive;<br>• cough or dyspnoea;<br>• chest infection not responding to appropriate first line antibiotics;<br>• abdominal pain or swelling;<br>• change in temperament or conscious level, or convulsions;<br>• new or progressive spinal or joint deformity	Any of the following:<br>• fever, wasting, lymphadenopathy;<br>• cough, tachypnoea, signs of respiratory distress;<br>• focal chest signs (e.g., bronchial breathing, crackles, wheeze, pleural rub, signs of pleural effusion);<br>• signs of pericardial effusion and/or congestive cardiac failure;<br>• abdominal mass, hepatomegaly, splenomegaly or ascites;<br>• lethargy, decreased conscious level, signs of meningism (photophobia,neck stiffness, Kernig's sign) or convulsions;<br>• spinal gibbus or enlarged non-tender joint;<br>• signs of tuberculin hypersensitivity (e.g., erythema nodosum, phlyctenular conjunctivitis)

Technical Appendix Table 2. Case definitions

Confirmed TB<br>• Disease at any site: Identification of M. tuberculosis complex (MTBC) from clinical specimens by culture or Xpert MTB/RIF assay, in the appropriate clinical context
Highly probable TB<br>• Disease at any site: positive microscopy for acid fast bacilli (AFB) but negative mycobacterial culture/PCR of clinical specimens in the appropriate clinical context;<br>• Disease at any site: histology of biopsy tissue showing caseating granulomata;<br>• Intra-thoracic TB: CXR appearances highly suggestive of active TB:<br>— non-pyogenic pleural effusion with no evidence of alternative cause<br>— cavitation associated with subacute/chronic pneumonia and no other identified cause of cavitation (e.g., Klebsiella or Staphylococcal sepsis)<br>— hilar/mediastinal lymph nodes plus a positive TST and no other identified cause;<br>• Miliary TB: miliary shadowing on CXR in an HIV un-infected child;<br>• TB Meningitis (TBM): clinical features of meningitis with CSF changes consistent with TBM (predominantly lymphocytic cellular infiltrate, protein concentration >0.8g/l, glucose concentration <2.2mmol/l, and no established alternative diagnosis);<br>• TB Lymphadenitis: cervical lymphadenopathy plus sinus formation or a positive TST;<br>• Abdominal TB: abdominal mass or ascites, with abdominal lymphadenopathy on ultrasound;<br>• Spinal TB: spinal gibbus in the absence of another obvious cause;<br>• Hypersensitivity reactions: erythema nodosum or phlyctenular conjunctivitis with chest radiograph evidence of primary TB
Confirmed or highly probable TB (CHPTB)<br>• Children who met the case definition for ‘Confirmed TB’ or ‘Highly Probable TB’
Possible TB<br>• Children treated for TB, but who did not meet the case definition for either ‘Confirmed TB’ or ‘Highly Probable TB’
All TB cases<br>• All children with ‘Confirmed TB’, ‘Highly Probable TB’, or ‘Possible TB’
Not TB (TB confidently excluded)<br>• All clinical features explained by a definitive alternative diagnosis, making TB highly unlikely; and/or insufficient clinical indication for a trial of TB treatment and no clinical deterioration during follow up in the absence of TB therapy
Not classifiable<br>• Children who did not meet criteria for confirmed, highly probable or possible TB, and in whom TB could not confidently be excluded, for example because they died or were lost to follow up

Technical Appendix Table 3. Estimated annual national case burden and incidence of childhood TB by age group (2010)

Age group	Child TB cases in the KHDSS (tb_{khdss})*	Ratio of child to adult cases in KHDSS (*r*)†	Child TB cases in Kenya (tb_{kenya})†	Kenya population (millions) (*14*)	Incidence per 100,000/y
0–4 y	86	86/678	11,401	6.2	184
5–9 y	31	31/678	4,110	6.0	69
10–14 y	9	9/678	1,193	5.4	22
<15 y	126	126/678	16,704	17.6	95

*tb_{khdss}, No. confirmed and highly probable TB cases after adjustment for the case detection rate
†*r*, Ratio of cases in age group to the total number of notified adult cases in the KHDSS (678)
‡tb_{kenya}, No. child TB cases in Kenya, estimated by multiplying number of notified adult cases in Kenya (89,883) by ratio (*r*) of child to adult cases in KHDSS.

Technical Appendix Table 4. Association of TB contact history with confirmed or highly probable TB among children investigated for TB at KCH and CPGH (Aug 2009–Jul 2011)

Characteristic	TB cases	Controls	Odds ratio for TB (95% CI)		p value
History of close TB contact					<0.0001
No history of close TB contact	87	1,074	1.0	(-)	
Any history of close TB contact	63	159	5.0	(3.4–7.3)	
Proximity of TB contact*					<0.0001
Contact outside the household	5	40	1.5	(0.6–4.0)	
Contact sleeps in same household	12	39	3.8	(1.9–7.5)	
Contact sleeps in same room	46	80	7.1	(4.6–10.8)	
Smear status of TB contact*					<0.0001
Smear negative	19	60	3.9	(2.2–6.8)	
Smear positive	38	70	6.7	(4.3–10.5)	
Smear status unknown	6	29	2.6	(1.0–6.3)	
Relationship of contact to child*†					<0.0001
Parent	40	75	6.6	(4.2–10.3)	
Grandparent	4	24	2.3	(0.8–6.7)	
Aunt or uncle	12	38	4.1	(2.0–8.3)	
Sibling	3	7	4.7	(1.1–20.8)	
Other	6	21	3.6	(1.4–9.3)	
No. close TB contacts*					<0.0001
1 close TB contact	58	147	4.9	(3.3–7.1)	
≥1 close TB contact	5	12	5.1	(1.8–14.9)	

*Compared with children who had no history of TB contact.
†Actual numbers of children presented, but weighted analysis used to derive odds ratios (see methods).

Technical Appendix Table 5. Population attributable fraction for a known household TB contact among KHDSS-resident children <5 y old (Aug 2009–Jul 2011)

Variable	Calculation
Numerators	
No. KHDSS-resident TB cases <5 y old with a known TB contact	$tb_{contacts} = 15$
No. KHDSS-resident TB cases <5 y old with no known TB contact	$tb_{non\text{-}contacts} = 15$
Denominators	
No. TB cases among KHDSS resident adults	$TB_{khdss} = 678$
Mean no. household contacts <5 y old per TB case	$\bar{C}_{household} = 362/195$
No. KHDSS resident contacts <5 y old of known TB cases	$N_{contacts} = TB_{khdss} \times \bar{C}_{household} = 1{,}259$
Person years observation among all KHDSS-resident children <5 y	$pyo_{total} = 89{,}503$
Person years observation among KHDSS-resident TB contacts <5 y	$pyo_{contacts} = N_{contacts} \times 2yrs = 2{,}518$
Incidence rates (per 100,000/year)	
TB incidence among child contacts of known TB cases	$I_{contacts} = \frac{tb_{contacts}}{pyo_{contacts}} = 596$
TB incidence among children with no known TB contact	$I_{non-contacts} = \frac{tb_{non-contacts}}{pyo_{total} - pyo_{contacts}} = 17$
Incidence rate ratio	$IRR = \frac{I_{contacts}}{I_{non-contacts}} = 35.1$
TB contact prevalence	
Prevalence of known TB contact among KHDSS-resident children <5 y	$p = \frac{N_{contacts}}{N_{total}} \equiv \frac{pyo_{contacts}}{pyo_{total}} = \frac{2{,}518}{89{,}503} = 2.8\%$
Population attributable fraction	
Population attributable fraction for a known TB contact	$PAF = \frac{p(IRR-1)}{p(IRR-1)+1} = 49\%$
